# Recurrent Urinary Bladder Paraganglioma

**DOI:** 10.1155/2010/912125

**Published:** 2010-06-27

**Authors:** Ali A. Al-Zahrani

**Affiliations:** Department of Urology, University of Dammam, Saudi Arabia

## Abstract

A 39-year-old male presented with recurrent attacks of painless haematuria. The patient has a history of partial cystectomy for bladder paraganglioma 10 years prior to the presentation. Imaging study and cystoscopic examination revealed a small anterior wall bladder tumor. The histological examination of the lesion confirmed that it was a urinary paraganglioma. Partial cystectomy was performed to this recurrent lesion. This case report stresses the importance of life-long follow-up of these lesions.

## 1. Introduction

Extra-adrenal paragangliomas account for approximately 15% of all pheochromocytomas. It arises from chromaffin cells of the paraganglionic system, and is located anywhere from the bladder to the base of the skull in association with the sympathetic chain [1].

In the urinary tract, paraganglia can persist around and within the bladder wall as well as along the ureter. Bladder paraganglioma constitutes less than 0.06% of all bladder tumors and less than 1% of pheochromocytoma [2]. The common presenting symptoms include paroxysmal hypertension, haematuria, postmicturition syncope, headache, sweating, and palpitation.

Surgical removal, either through partial cystectomy or transurethral resection, is still the gold standard of treatment. The current case report stresses the importance of the life-long regular follow-up after the surgical management to detect possible remote recurrence.

## 2. Case Report

**Figure 1:**
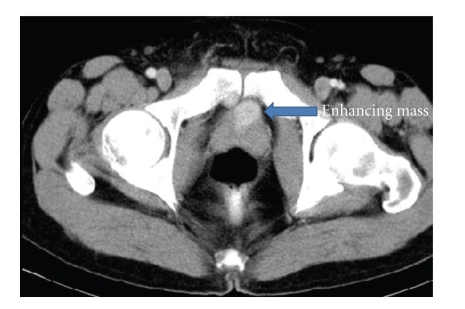
Axial CT scan of the pelvis after IV contrast: oval shape well-defined homogenous small mass at the bladder neck which shows intense enhancement after contrast.

A 39-year-old male presented with recurrent attacks of gross painless haematuria for 5 months. The patient has a history of partial cystectomy for paraganglioma 10 years prior to the presentation. The pathological report stated that the surgical margin was negative. The urine catecholamine level postresection was normal. He was followed for one year postoperatively with cystoscopic examination without any sign of recurrence. The patient does not have similar history in the family. Regarding his second presentation, the patient had no attacks of headache, sweating, palpitation, anxiety, or postmicturition syncope. His blood pressure was within normal range. Urine analysis revealed red blood cells in large quantity. Plasma metanephrines and urine vanillylmandelic acid (VMA) levels were within normal limits. Abdominal CT scan showed a 3 × 2 cm oval-shaped homogenous anterior bladder wall mass. The mass showed intense uniform enhancement with contrast study. There was no associated lymphadenopathy or invasion to the surrounding pelvic structures (Figure 1). 

**Figure 2:**
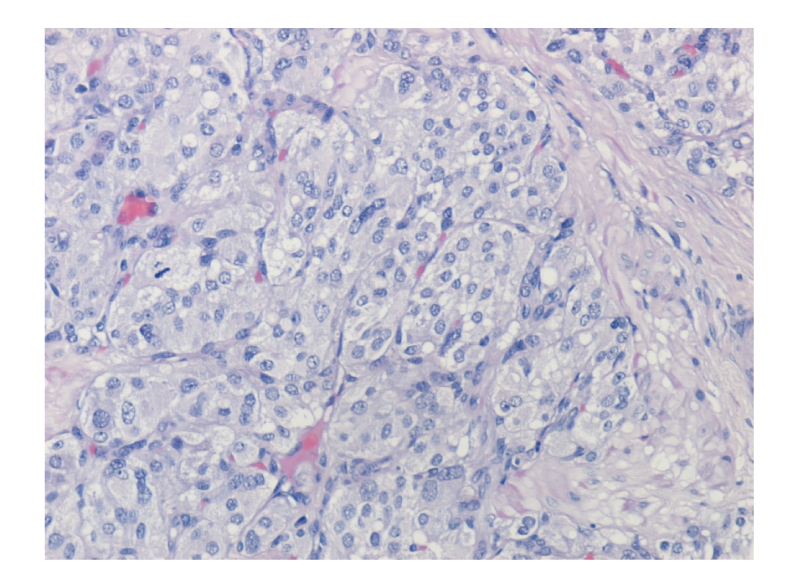
The tumor cells show the characteristic zellballen cells, with abundant eosinophilic cytoplasm and an ill-defined wall separated by fibrovascular stroma. Mitotic figures are not observed. Hematoxylin-Eosin Stain (X400).

**Figure 3:**
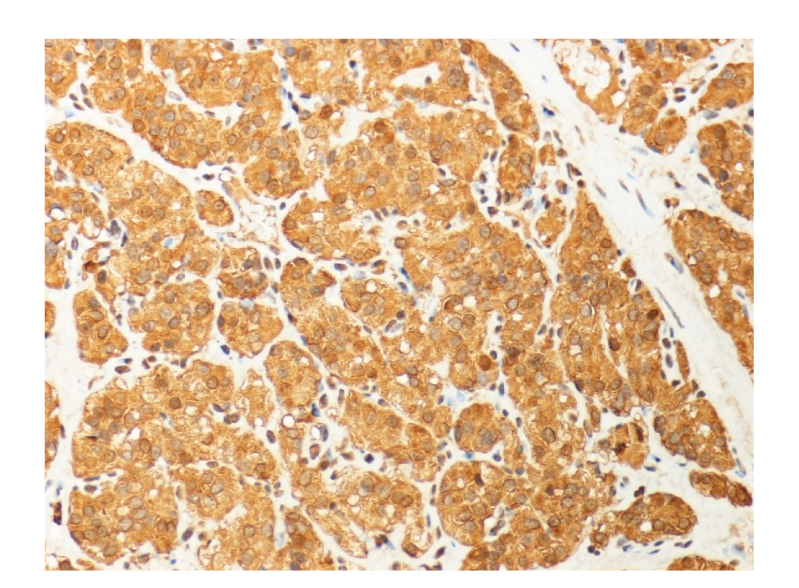
Immunostaining for chromogranin A showed strong and diffuse positivity in the tumor cell cytoplasm (X400).

Because of the history of bladder paraganglioma, the patient was started on *α* adrenergic blockade. Cystoscopic examination under spinal anesthesia showed a small exophytic yellowish submucosal mass in the left anterior wall near the bladder neck. Histological analysis revealed a highly vascular neoplasm covered by normal urothelium. Tumor cells were backed into small nests surrounded by a fibrovascular network. The cytoplasm was eosinophilic with indistinct borders (Figure 2). Immunostains were positive for chromogranin A, neurone-specific enolase, S-100 protein and sinaptophysin, while immunostains for epithelial differentiation (using antikeratin monoclonal antibody) were negative (Figure 3). These findings were consistent with paraganglioma of the bladder. There was no change in blood pressure during cystoscopy or during manipulation of the tumor. Partial cystectomy was carried out with uneventful postoperative course. The surgical margin of the removed specimen was negative.

Postoperative hormone levels, including plasma metanephrines, and urinary VMA, were normal. Iodine-131-*m*–iodobenzylguanidine (^131^I-MIBG) scintigraphy and abdominal CT scan 12 months after the procedure showed no recurrence.

## 3. Discussion

Bladder pheochromocytoma occurs more frequently in women than in men, and mainly during the third decade of life [3]. The tumor is usually benign, only 15–20% being malignant [4]. The presenting symptoms usually result from excessive catecholamine secretion. The patient typically suffers from hypertensive crises that may be accompanied by headache, palpitations, hot flushes, and sweating. These crises are mainly provoked by micturition, overdistention of the bladder, defecation, sexual activity, ejaculation, or bladder instrumentation. Haematuria is the presenting complaint in about 60% of reported cases. It has been estimated that only 17% of bladder pheochromocytomas patient are hormonally nonfunctional (paraganglioma) [5].

At present, the best imaging method to detect and follow patients with paragangliomas is uncertain. Anatomical imaging with CT scan and MRI can usually detect most of these lesions. MRI using various sequences has been proven to be superior to CT scan [1]. In addition, intravenous contrast of the CT scan can provoke the catecholamine release [6]. Functional imaging that specifically targets the catecholamine synthesis, storage, and secretion pathway is helpful in patients with paraganglioma especially after surgical removal or in detection of metastasis. These imaging modalities include ^123^I-MIBG, ^18^F-FDA, ^18^F-FDOPA, ^18^F-FDG,^ 11^C-epinephrine, and ^11^C-hydroxyephedrine. ^123^I-MIBG is preferred over ^131^I-MIBG because of higher sensitivity, lower radiation exposure, and improved imaging quality [7]. Timmers et al. reported 97% sensitivity of ^18^F-FDG in patients with metastatic paraganglioma [8]. One of the drawbacks of the functional studies is that the materials tend to accumulate in the bladder and hide the bladder lesion. Some reports had recommended bladder irrigation to delineate the bladder lesion [9]. 

The patient had his first paraganglioma removed at age of 29 which raise the suspicion of familial form of the disease. Recent data have suggested that between 25%–30% of these tumors might be familial in nature [10]. Germline mutations in NF1, ret, VHL, SHDB, SDHC, and SDHD had been associated with paraganglioma [11]. Korpershoek et al. recommended genetic testing for patients with extra-adrenal, bilateral, or multiple pheochromocytomas regardless of age, because it is known that such presentations frequently occur in the context of a heritable tumor syndrome [11].

Assessments of plasma and/or urine catecholamine levels are very important in these patients even if they are asymptomatic. It is crucial in the initial assessment and in the follow up afterward. Lenders et al. reported that plasma metanephrines are more sensitive and specific than urinary metanephrines for these lesions [12]. On the other hand, we should be aware that medications, including tricyclic antidepressants, decongestants, amphetamines, antipsychotic medications, reserpine, levodopa, ethanol, and acetaminophen, can increase both urine and plasma catecholamine measurements and cause false positive tests [13].

Cystoscopic appearance of a yellow, submucosal tumor should raise the suspicion of a bladder pheochromocytoma. Precautions should be taken to avoid potentially lethal transient hypertensive crises during tumor manipulation. Preoperative alpha blockade is used to counteract the effects of the elevated catecholamines during surgical manipulation. Before tumor removal, beta blockers can be initiated to protect against cardiovascular abnormalities like tachycardia or arrhythmias.

Treatment of bladder paraganglioma requires total excision of the tumor by partial cystectomy. Transurethral resection is usually inadequate because the majority of tumors extend in the deep layers of the detrusor muscle. Total cystectomy is reserved for large lesions when bladder preservation is impossible or in the presence of lymph node metastasis. Pelvic lymph node dissection has been recommended in order to exclude metastatic disease in all cases of bladder paraganglioma [5]. The prediction of the biological behavior of a bladder paraganglioma cannot be based solely on the histopathological findings. Some criteria have evolved to suggest malignancy behavior which includes necrosis, angiolymphatic invasion, high mitotic index, absence of hyaline bodies, p53 alteration, and DNA ploidy analysis. However the diagnosis of a malignant tumor is difficult and is often proved clinically through presence of metastases [4]. Recurrence of the disease at the site of resection should not be considered as evidence of malignancy [14]. Bladder paraganglioma is a chemo-resistant and radio-resistant tumor, though such regimens were used in inoperable cases with relative efficacy [15].

Life-long follow-up is necessary to detect late recurrences [14]. Follow up should be regular and include cystoscopic examination, plasma or urinary catecholamines levels and imaging study (CT scan and  ^ 123^I-MIBG scintiscan). There is no consensus about the frequency of these measures; however, we suggested that there should be at least an annual follow-up for these patients if they are asymptomatic or whenever clinically indicated.

## Abbreviations

CT: Computerized tomography
I-MIBG: iodine-metaiodobenzylguanidine
VMA: vanillylmandelic acid
MRI: Magnetic resonance imaging
FDA: fluorodopamine
FDOPA: florodopaphenylalanine
FDG: fluoro-2-deoxy-D-glucose
NF1: Neurofibromatosis
Ret: Retina
VHL: Von Hippel-Lindau
SHD: succinate dehydrogenase
DNA: deoxyribonucleic acid.

## References

[1] Whalen RK, Althausen AF, Daniels GH (1992). Extra-adrenal pheochromocytoma. *Journal of Urology*.

[2] Thrasher JB, Rajan RR, Perez LM, Humphrey PA, Anderson EE (1993). Pheochromocytoma of urinary bladder: contemporary methods of diagnosis and treatment options. *Urology*.

[3] Bonacruz Kazzi G (2001). Asymptomatic bladder phaeochromocytoma in a 7-year-old boy. *Journal of Paediatrics and Child Health*.

[4] Piédrola G, López E, Rueda MD, López R, Serrano J, Sancho M (1997). Malignant pheochromocytoma of the bladder: current controversies. *European Urology*.

[5] Das S, Bulusu NV, Lowe P (1983). Primary vesical pheochromocytoma. *Urology*.

[6] Bessell-Browne R, O’Malley ME (2007). CT of pheochromocytoma and paraganglioma: risk of adverse events with IV administration of nonionic contrast material. *American Journal of Roentgenology*.

[7] Lynn MD, Shapiro B, Sisson JC (1985). Pheochromocytoma and the normal adrenal medulla: improved visualization with I-123 MIBG scintigraphy. *Radiology*.

[8] Timmers HJLM, Kozupa A, Chen CC (2007). Superiority of fluorodeoxyglucose positron emission tomography to other functional imaging techniques in the evaluation of metastatic SDHB-associated pheochromocytoma and paraganglioma. *Journal of Clinical Oncology*.

[9] Kosuda S, Kison PV, Greenough R, Grossman HB, Wahl RL (1997). Preliminary assessment of fluorine-18 fluorodeoxyglucose positron emission tomography in patients with bladder cancer. *European Journal of Nuclear Medicine*.

[10] Erlic Z, Neumann HPH (2009). When should genetic testing be obtained in a patient with phaeochromocytoma or paraganglioma?. *Clinical Endocrinology*.

[11] Korpershoek E, Petri B-J, Van Nederveen FH (2007). Candidate gene mutation analysis in bilateral adrenal pheochromocytoma and sympathetic paraganglioma. *Endocrine-Related Cancer*.

[12] Lenders JWM, Pacak K, Walther MM (2002). Biochemical diagnosis of pheochromocytoma: which test is best?. *Journal of the American Medical Association*.

[13] Adler JT, Meyer-Rochow GY, Chen H (2008). Pheochromocytoma: current approaches and future directions. *Oncologist*.

[14] Maddocks RA, Fagan WT (1976). Paraganglioma of bladder with recurrence ten years later. *Urology*.

[15] Naguib M, Caceres M, Thomas CR, Herman TS, Eng TY (2002). Radiation treatment of recurrent pheochromocytoma of the bladder: case report and review of literature. *American Journal of Clinical Oncology*.

